# Health Benefits of Lactic Acid Bacteria (LAB) Fermentates

**DOI:** 10.3390/nu12061679

**Published:** 2020-06-04

**Authors:** Harsh Mathur, Tom P. Beresford, Paul D. Cotter

**Affiliations:** 1 Food for Health Ireland, Teagasc Food Research Centre, Moorepark, Fermoy, P61 C996 Co. Cork, Ireland; harsh.mathur@teagasc.ie; 2 Teagasc Food Research Centre, Moorepark, Fermoy, P61 C996 Co. Cork, Ireland; 3 APC Microbiome Ireland, University College Cork, T12 YN60 Co. Cork, Ireland

**Keywords:** fermentates, lactic acid bacteria (LAB), health effects, gut, immunomodulation, antimicrobial

## Abstract

Consuming fermented foods has been reported to result in improvements in a range of health parameters. These positive effects can be exerted by a combination of the live microorganisms that the fermented foods contain, as well as the bioactive components released into the foods as by-products of the fermentation process. In many instances, and particularly in dairy fermented foods, the microorganisms involved in the fermentation process belong to the lactic acid group of bacteria (LAB). An alternative approach to making some of the health benefits that have been attributed to fermented foods available is through the production of ‘fermentates’. The term ‘fermentate’ generally relates to a powdered preparation, derived from a fermented product and which can contain the fermenting microorganisms, components of these microorganisms, culture supernatants, fermented substrates, and a range of metabolites and bioactive components with potential health benefits. Here, we provide a brief overview of a selection of in vitro and in vivo studies and patents exclusively reporting the health benefits of LAB ‘fermentates’. Typically, in such studies, the potential health benefits have been attributed to the bioactive metabolites present in the crude fermentates and/or culture supernatants rather than the direct effects of the LAB strain(s) involved.

## 1. Introduction

Several studies have reported the health benefits of fermented foods. Fermented foods typically contain microorganisms of a generally regarded as safe (GRAS) status that can produce a number of beneficial by-products/metabolites such as antimicrobial peptides (e.g., bacteriocins), ethanol, organic acids, fatty acids, carbon dioxide, amongst others [1,2]. Fermentations typically involve the utilization of compounds in the raw substrate by microorganisms, such as lactic acid bacteria (LAB) and yeasts [1]. The Lactic acid bacteria (LAB) are a group of bacteria that include genera such as *Lactobacillus*, *Lactococcus*, *Pediococcus*, *Enterococcus*, and *Streptococcus* and are frequently found in dairy fermented foods [3]. Aside from dairy fermented foods, LAB are also found in fermented vegetables [4,5], fermented meat [6,7], and fermented cereals [8,9,10]. LAB-driven fermentations often yield by-products with bioactivity and a diverse range of health-promoting effects, including protection against infectious agents, immunomodulatory effects, anti-allergenic effects, anti-obesity effects, anti-oxidant effects, enhancing the bioavailability of vitamins/minerals, anti-anxiety effects, among others [11,12,13,14,15,16,17,18,19]. For instance, one meta-analysis has highlighted the effects of consuming LAB fermented foods and positive impacts on weight maintenance [20], while separate studies have demonstrated that consumption of fermented yogurts and dairy foods can attenuate the likelihood of developing cardiovascular disease (CVD) and type 2 diabetes mellitus (T2DM) [21,22,23,24]. Fermented milk has the potential to ameliorate the mechanisms involved in glucose metabolism and reduce muscle pain associated with resistance exercise [25], while other fermented foods such as kimchi have demonstrated anti-obesity and anti-diabetic effects [26,27]. The mechanisms involved in insulin-mediated uptake of glucose can be compromised in damaged muscles following exercise [28,29]. However, the above-mentioned study by Iwasa et al. reported that fermented milk can result in an improvement in the ability of damaged muscles to utilize glucose [25]. While there are limited clinical data in the area, studies have suggested that fermented foods can have a positive impact on inflammatory bowel disease (IBD) symptoms [30]. Indeed, due to the LAB-associated fermented foods, the gut microbiota can also be altered in a positive manner and such foods can thereby assist in the resolution of IBD [30,31]. It has been suggested that fermented dairy products have the ability to positively modulate the gut microbiota, either by stimulating the proliferation of beneficial microbes and/or by introducing novel species into the gut [32,33,34]. Other investigators have studied the hypocholesteremic and anti-cancer properties of these LAB-associated fermented as well as non-fermented milks [35]. Finally, several recent studies have indicated that the consumption of fermented foods can be associated with improvements in mood and cognitive function [36,37,38].

As mentioned, a range of bioactive compounds can potentially be produced during LAB fermentation processes. One example of such bioactive components includes angiotensin converting enzyme (ACE)-inhibitory peptides. More specifically, certain peptides associated with fermented milks, such as valyl-prolyl-proline (VPP) and isoleucyl-prolyl-proline (IPP), display anti-hypertensive characteristics and could potentially be harnessed as alternative/adjunct therapeutic options to manage hypertension [39,40,41,42,43]. Some LAB strains can also produce exopolysaccharides (EPS) during fermentation [44] and such EPS have shown anti-diabetic, cholesterol-reducing, anti-oxidant, and immunomodulatory effects in a number of studies [45,46,47,48]. The cholesterol-lowering properties of these EPS from LAB are likely to be elicited by decreasing cholesterol absorption in the gut, stimulating the release of bile acids and by binding cholesterol [45,49,50]. Aside from their cholesterol-lowering properties, EPS are also critical in host–microbe interactions by assisting the microbes to colonize in the gut, as well as exerting immunomodulatory effects [51,52]. Several of these LAB strains can also produce enzymes, such as amylase, protease, lipase, and glucoamylase, during fermentation and thus assist in breaking down complex inedible substrates, thereby facilitating increased nutrient absorption [53,54,55]. Other gut-associated benefits of LAB-derived fermented foods include an enhanced capacity to absorb minerals due to the breakdown of phytic acid, addressing lactose intolerance, inhibition of antibiotic-associated diarrhea (AAD), amongst other benefits. In vitro assays have also indicated that fermented food-associated bioactives can exert anti-tumor effects on the gastric cancer cell line, SGC7901 [56]. Finally, antimicrobial end-products such as bacteriocins (ribosomally synthesized peptides) can also be produced during the fermentation process [57]. For more information regarding the health benefits of fermented foods, the reader is referred to comprehensive reviews by Marco et al. [1] and Macori et al. [2].

While it has been established in several studies and summarized in numerous reviews that consumption of fermented foods and their associated microorganisms confers health benefits on the host, for the purposes of this mini-review, we specifically focus on any such patents and studies conducted with LAB ‘fermentates.’ When mentioning the term ‘fermentates’ hereafter, we refer to compositions/formulations which are typically composed of LAB cells either in an active or inactive state, along with the culture supernatants and fermentation media, which may contain metabolites and bioactive components, which are often responsible for conferring the health benefits mentioned herein. Any such fermentates typically undergo freeze-drying or spray-drying processes. Some potential benefits of using fermentates include an extended shelf life, the possibility to use non-replicating bacterial cells, as well as using fermentates as food ingredients, whilst simultaneously harnessing the specific health benefits of fermented foods. Here, we summarize the studies and patents specifically related to the health benefits of LAB ‘fermentates.’ One key focus of the review is to determine to what extent the health attributes associated with fermented foods can be found in fermentates.

## 2. Studies and Patents Relating to LAB Fermentates and Effects on Overall Health

### 2.1. LAB Fermentates and Gut Health

Thus far, there have been a very limited number of studies conducted specifically investigating the effects of LAB fermentates on gut health. Nonetheless, in one relatively recent patent, Fasano and Budelli reported the effects of fermentates produced with the LAB strain *Lactobacillus paracasei* CBA L74 (LMG P-24778) with respect to alleviating gluten-associated disorders, such as gluten sensitivity and celiac disease (Patent application number 18157990.5)(Patent number EP 3 351 554 A1) [58]. The inventors of the patent noted that compositions containing the strain itself, the cell-free supernatants, fermentates or metabolites of the culture somehow block or hinder gluten or gluten-associated polypeptides from traversing the intestinal mucosa. By doing so, the negative effects of gluten and gluten-associated peptides on patients with such disorders are somewhat mitigated. More specifically, the metabolites associated with fermentates of this strain decreased the influx of gliadin peptides into human intestinal epithelial cells. Undigested gliadin peptides typically trigger inappropriate innate as well as adaptive T cell-mediated immune responses in the celiac small intestinal mucosa, leading to the symptoms associated with gluten-associated diseases [59,60].

In addition to the above-mentioned effects, Fasano and Budelli found that the cell-free supernatants of CBA L74 reduce the entry of dextran into cells [58]. The inventors postulate that the positive effects exerted by fermentates derived from the CBA L74 strain can be ascribed to fermentation-derived metabolites such as acetic acid, butyric acid, lactic acid, acetaldehyde, acetoin, diacetyl, butanediol, ethanol, acetate, formate, either individually or in combination with each other, but there is limited evidence provided to support this hypothesis or how these metabolites could reduce the uptake of molecules. Although they were not fully characterized in the work described in the patent, the metabolite(s) responsible for attenuating the entry of gliadin into cells is likely to be found in the culture cell-free supernatants, culture media, or fermented media [58].

With regards to other studies relating to the effects of dairy fermented foods on gastrointestinal tract (GIT) health and nutrient absorption, Saqib and co-workers [61] have shown that *Propionibacterium spp*. can produce β-galactosidase (lactase), which breaks down lactose, thereby alleviating symptoms associated with lactose intolerance [18,61,62]. Others have referred to the ability of *Bifidobacterium* strains to assist in the digestion of lactose as well by producing lactic acid, thus resolving symptoms of lactose intolerance [63]. Ultimately, the ability to break down lactose is a trait likely shared to some extent by all LAB and bifidobacteria. Fermented foods encompassing *Lactobacillus* and *Bifidobacterium* strains can contain a catalog of B vitamins as well [64,65,66]. Finally, a study by Rodríguez et al. has highlighted the gastro-protective effects of LAB-derived fermented foods [67]. The study highlighted the effectiveness of milks fermented with EPS-producing *Streptococcus thermophilus* strains CRL 804 and CRL 1190 in preventing chronic gastritis, using BALB/c mouse models. Administration of milk fermented with strain CRL 1190 prevented the development of gastritis in mice which were subsequently given acetylsalicylic acid, and resulted in gastric mucosa histology akin to healthy mice. In addition, a significant increase in IL-10(+)-producing cells and a significant decrease in IFNgamma (+)-producing cells were elicited by the fermented milk [67].

### 2.2. Effects of LAB Fermentates on Appetite Modulation and Weight Management

In relation to weight gain and modulation of appetite, studies conducted as part of the research group, Food for Health Ireland (FHI), included the creation of a bank of a number of milk fermentates using a range of LAB strains. Reconstituted skim milk (RSM) was utilized as a substrate for fermentation since it is suitable for growing several LAB strains. As part of the study, several different LAB strains belonging to the genera *Lactobacillus*, *Lactococcus*, *Pediococcus*, and *Leuconostoc* underwent the fermentation process in 10% as well as 30% RSM once their ability to grow in milk was established. The conditions employed during fermentations were such that they were suitable for commercial scale up. As expected, most of the LAB strains tested were able to grow during the fermentation process, and this was confirmed by increases in cell numbers, with concomitant reductions in pH. A number of in vitro assays were conducted with these LAB fermentates, including testing for the suppression of pancreatic lipase (PL). PL is the main enzyme which hydrolyzes dietary fat into monoglycerides and free fatty acids [68,69,70]. The various fermentates were screened for their ability to suppress PL, with a view to decreasing fat absorption in the gut. It was shown that some of the fermentates were able to reduce PL activity by up to 50%, compared to a 70% inhibition elicited by the commercial medication Orlistat. The degree of PL inhibition varied with the particular species and strain of bacteria used, as well as the fermentation conditions employed [71].

A major hormone, which is associated with suppression of appetite, is glucagon-like peptide-1 (GLP-1) and it has been suggested that induction of GLP-1 results in decreases in appetite and consequent intake of food [72,73,74]. A study by Chaudhari and co-workers investigated the activity of *Lactobacillus helveticus* NCDC 288 and NCDC 292-derived fermentates with respect to the expression of cholecystokinin (CCK), proglucagon, and pro-gastric inhibitory peptide (pro-GIP) by real-time polymerase chain reaction (PCR) [75]. The authors noted that the *Lb. helveticus* fermentates stimulated the expression of CCK, pro-GIP, proglucagon, and GLP-1 from STC-1 cells. More specifically, CCK, proglucagon, and pro-GIP gene expression was triggered by hydrolysates of whey protein and casein protein, and this expression in turn stimulated the STC-1 cells (pGIP/Neo) to secrete GLP-1. However, the authors were unable to recover one single active peptide from milk fermented with *Lb. helveticus*. Nonetheless, this in vitro study showed that the fermentates from the *Lb. helveticus* strains used have the ability to stimulate GLP-1 production and thus warrant further investigation in in vivo trials as effective therapeutics for T2DM [75].

### 2.3. Immunomodulatory Effects of LAB Fermentates

Overall, there are a very limited number of studies conducted investigating LAB fermentates and immunomodulation. Nonetheless, a patent outlining the immunomodulatory effects of fermentates of the same *Lb. paracasei* CBA L74 strain mentioned previously was filed by Budelli and co-workers (Patent application number 18166007.7) (Patent number EP 3 366 143 A1) [76]. In this patent, the inventors outlined methods of obtaining the fermentates comprising the CBA L74 strain in detail and reported the outcomes of various in vitro and in vivo assays conducted to evaluate the immunomodulatory effects of such fermentates.

Aside from the methodology outlining the composition of the fermentates, the inventors described the efficacy of *Lb. paracasei* CBA L74 fermented foods in modulating the mucosal immune system. They demonstrated that exposure to CBA L74 cells induced increased production of the anti-inflammatory cytokine IL-10 in in vitro assays involving co-culture of dendritic and Caco2 cells. In animal studies, they demonstrated that administration of *Lb. paracasei* CBA L74 resulted in reduced titers of the pro-inflammatory mediator IL-1β in the mucosa of mice. They also reported an up-regulation and increase in production of Toll-like receptor 2 (TLR2) and TLR4, which are both critical players in the immune system and are involved in the recognition of conserved microbial structures. Feeding skim milk fermented with *Lb. paracasei* CBA L74 to mice resulted in a significant elevation of IL-10 in the intestinal mucosa with only a modest increase in IL-1β titers; however, only minor changes in levels of expression of TRL2 and TRL4 were observed. Thus, these data indicate that *Lb. paracasei* CBA L74 and milk fermentates made using this strain can impact on the expression of different cytokines and receptors involved in the innate immune system. Budelli et al. further hypothesize that foods fermented with this CBA L74 strain may confer a health benefit via competitive exclusion of certain pathogens, in addition to forming a protective barrier across the GIT mucosa. The *Lb. paracasei* CBA L74 cells can be in either an active or inactive form in the fermentate formulation [76]. Indeed, metabolites present in the fermentates or culture supernatants, such as acetic acid, butyric acid, and lactic acid, synthesized by probiotics during fermentation, prior to heat inactivation, have also been reported to confer health benefits on the host in other studies [77,78,79,80,81]. Thus, it may be the case that the mucosal immune system can be upregulated upon exposure to fragments from the bacterial cell wall, unmethylated CpG dinucleotides, and/or other proteins as well as lipids or carbohydrates associated with the *Lb. paracasei* CBA L74 cells [76]. The CBA L74 strain can also be included in fermented foods, and cell numbers in such fermented foods can vary between 1 × 10^2^ colony forming units (cfu)/g and 1 × 10^12^ cfu/g. The potential applications of such fermented foods include alleviating a wide range of GIT disorders such as food allergies, bacterial or viral infections, Crohn’s disease, necrotizing enterocolitis, amongst others [76].

### 2.4. In Vivo Studies Investigating the Anti-Infective Effects of LAB Fermentates

A few groups have investigated the anti-infective properties of LAB fermentates by conducting in vivo studies. For instance, a patent filed by Fasano and Rescigno, utilizing the same *Lb. paracasei* CBA L74 strain mentioned previously, described the effects of fermentates of the strain based on the results of an animal trial (Patent application number 18209662.8)(Patent number EP 3 470 074 A1) [82]. The inventors described a decrease in the severity of an infection in offspring of lactating mice as a result of the fermentate, and indicated that similar fermentates could potentially prove effective for humans as well. Similar to other inventions, the optimum formulation consists of foods fermented by this probiotic organism. The work outlined in the patent was conducted with pregnant or lactating animals and it was determined that rice fermented with *Lb. paracasei* CBA L74 conferred a health benefit when given to pregnant and lactating mice, in terms of attenuating the severity of infections caused by *Cronobacter sakazakii*. Consumption of milk fermented with this strain by pregnant mice also resulted in a protective effect against other pathogens such as *Salmonella typhimurium* FB62, *Listeria monocytogenes*, and *Escherichia coli*. In order to tackle such infections in offspring of lactating mothers, the results of the animal trial indicated that administration of the CBA L74-associated fermentate or fermented food to lactating mothers is effective both prenatally, as well as postnatally [60,82].

A separate study conducted by Casey et al. reported the outcomes of an animal trial in which the authors administered a mixture of five LAB strains as milk fermentates or milk suspensions to weaned pigs for a period of 30 days [83]. These five LAB strains included *Lactobacillus pentosus* DPC6004, *Lactobacillus salivarius* subsp. *salivarius* DPC6005, *Pediococcus pentosaceus* DPC6006, *Lactobacillus murinis* DPC6002, and *Lb. murinis* DPC6003. After six days of administration of the LAB fermentates, the pigs were infected with *Salmonella serovar Typhimurium* and the overall health and microbiological analysis of their feces was conducted. The investigators determined that *Salmonella* numbers in feces were significantly decreased and overall health of the animals improved as a consequence of administration of the fermentates derived from the five LAB strains [83]. As part of the study, 10% RSM supplemented with yeast extract was utilized to grow four of the five LAB strains, while RSM without yeast extract was used to grow the remaining *Lb. salivarius* DPC6005 strain. The fermentates from the five LAB strains were mixed together in order to feed the pigs for the trial which were fed a total of approximately 4 × 10^10^ cfu/day from the fermentates throughout the course of the trial. The authors also determined that the cell numbers of each of the five strains contained in the mixed fermentate were approximately equal to each other. Although not statistically significant, pigs fed with the LAB-derived fermentate managed to gain more weight (mean increase of 632.4 ± 34.5 g/day) than the control group. The authors also found that from the groups that were fed the LAB suspension or LAB fermentate, only 6/50 fecal samples collected from the pigs post-*Salmonella* infection were positive for diarrhea. This compared to 13/25 diarrhea-positive samples from the control treatment group. Average *Salmonella* numbers present in feces from the LAB fermentate group were lower (2.04 log_10_ cfu/g feces), compared to the control treatment group (3.51 log_10_ cfu/g), eight days post-infection. At 15 days post-infection, the corresponding average *Salmonella* numbers for the fermentates treatment group of 1.42 log_10_ cfu/g feces, were significantly lower than the 3.68 log_10_ cfu/g for the control treatment group [83]. Finally, one study by Sreekumar and Hosono showed that skim milk medium fermentates containing *Lb. acidophilus* SBT2074 inhibited *Escherichia coli* 3544, and thus could have potential for individuals with GIT infections with coliforms such as *E. coli*. [84].

A separate animal trial describing fermentates containing the bacteriocin, lacticin 3147, was conducted with a view to treating bovine mastitis [85]. Lacticin 3147 is a relatively broad spectrum two-component bacteriocin belonging to the lantibiotic subclass [86]. As part of the bovine mastitis trial, Klostermann et al. developed a natural teat dip utilizing a fermentate encompassing viable cells of *Lactococcus lactis* DPC3251, which produce lacticin 3147 [85]. Initial in vitro trials as part of the study involved introducing 10^5^ cfu/mL of the mastitis-causing pathogens, *Streptococcus uberis* Diernhofer 1932^AL^, *Streptococcus dysgalactiae* subsp. *dysgalactiae* M, and *Staphylococcus aureus* DPC5246 into the lacticin 3147-containing fermentate. It was shown that *Strep. dysgalactiae* subsp. *dysgalactiae* M and *S. aureus* DPC5246 were undetectable after 15 and 30 min respectively, whereas *Strep. uberis* Diernhofer 1932^AL^ numbers were decreased 100-fold after 15 min as a result of exposure to the fermentate containing lacticin 3147. Following this, as part of the in vivo trials in the study, teats of lactating dairy cows were coated with each of the pathogens and subsequently dipped into the teat dips containing the lacticin fermentate. *Strep. dysgalactiae* was decreased by 97%, *Strep. uberis* by 90%, and *S. aureus* by 80% after a 10 min dip contact time, thus demonstrating the efficacy of teat dips containing lacticin fermentates [85].

In order to prepare the teat dip, the *L. lactis* DPC3251 strain was first grown in microbiological media overnight and then subsequently in 10% RSM for 20 h at 30 °C [85]. This fermentate in RSM was kept at 4 °C and utilized as the DPC3251 teat dip for the assays used in the study. In addition, as a negative control, a fermentate from the bacteriocin-negative strain *L. lactis* DPC5399 was prepared under the same conditions. The consistencies and pH of fermentates derived from both the DPC5321 and DPC5399 strains were very similar [85]. Furthermore, as part of the study, the authors evaluated the stability of the DPC3251-derived fermentate when stored at 4 °C, with regards to maintenance of bacteriocin antimicrobial activity. Encouragingly, the antimicrobial activity of the bacteriocin was retained at 53% of its baseline level, even three weeks after storage of the fermentate at 4 °C [85].

### 2.5. In Vitro Studies Investigating the Effects of LAB Fermentates on Food-Borne Pathogens

A few studies have reported the antimicrobial effects of LAB fermentates on food-borne pathogens. Examples of such cases include the development of whey powder-based spray-dried formulations containing the bacteriocin lacticin 3147, produced by the LAB, *L. lactis* DPC3147 [86,87]. In the initial study by Morgan and co-workers, which described the optimization of the formulation, the fermentate containing lacticin 3147 was prepared by inoculating a 170 L volume of demineralized whey powder (containing approximately 10% solids) with a 1% inoculum of *L. lactis* DPC3147, and fermentation took place for 24 h with pH constant at 6.5 [87]. The fermentate was pasteurized at 72 °C for 15 s prior to evaporation to 40% total solids. Finally, spray drying was employed to further concentrate the fermentate and yield a powder containing bioactive lacticin 3147. After spray drying, the lacticin 3147-containing fermentate concentrated powder was evaluated for its ability to inhibit the pathogens *S. aureus* 10 and *L. monocytogenes* Scott A in buffer at pH 5 and pH 7. Furthermore, the powder was tested for its ability to target *L. monocytogenes* Scott A in infant milk formulations. Addition of approximately 10^8^ cfu of mid-exponential phase *L. monocytogenes* cells in 10% lacticin-enriched powder led to an approximate 3-log decrease in viable cells within 3 h at 30 °C at both pH 5 and pH 7. In contrast, the lacticin 3147-enriched powder was less effective at pH 5 against *S. aureus* 10, as addition of approximately 10^7^ cfu mid-exponential phase cells in a 15% solution of the lacticin powder merely resulted in a log decrease within 3 h. However, there was a 3-log decrease at a neutral pH within 3 h. Finally, a 99% decrease in *L. monocytogenes* numbers within 3 h was noted when tested in infant milk formulations supplemented with the lacticin powder [87]. As part of a follow-up study, the authors assessed the efficacy of this fermentate in targeting the food-borne pathogens *Bacillus cereus* and *L. monocytogenes.* The fermentate elicited a 99.9% and 85% decrease in *L. monocytogenes* numbers within 2 h in natural yogurt and cottage cheese respectively. Furthermore, *B. cereus* counts were decreased by 80% in soup within a 2 h timeframe in the presence of 1% lacticin 3147 powder [88].

With respect to other studies assessing fermentates derived from lantibiotic-producing LAB strains, Field et al. compared the efficacies of nisin A and nisin V fermentates independently and in combination with essential oils with a view to targeting *L. monocytogenes* [89]. Nisin A is a single peptide lantibiotic and is used worldwide as a food preservative [90]. Nisin V is a bioengineered derivative (with a methionine to valine substitution at residue 21) of the parental nisin A peptide, and has demonstrated increased potency against a number of target strains [89]. Field and co-workers found that the nisin V-containing fermentate, when combined with cinnamaldehyde and carvacrol, was particularly effective at targeting *L. monocytogenes*. Importantly also, it was established that the increased potency and specific activity of nisin V compared to nisin A was maintained in fermentate form in growth media, as well as in food matrices [89].

Another bacteriocin which has been prepared in fermentate form includes pediocin PA-1. Pediocin PA-1 is a class II bacteriocin, produced by the LAB, *Pediococcus acidilactici*, and is particularly potent against *L. monocytogenes* [91,92,93]. Pediocin PA-1 can be prepared as a crude fermentate, an example of which includes MicroGARD, and is commercially used as a food biopreservative and in particular to reduce *L. monocytogenes* populations in food [94,95,96]. MicroGARD fermentates are available in many forms from the company DuPont, including products ranging from MicroGARD 100, 200, 300, 400, 520, 730, CS1-50, CM1-50. There are slight variations in the benefits and applications of these different products. For instance, MicroGARD 300, 400, 520, 730, CS1-50, and CM1-50 have the ability to target Gram-positive bacteria, whereas MicroGARD 100, 200, 400, and 730 are effective at inhibiting Gram-negative bacteria (DuPont). These MicroGARD fermentates can be utilized in a range of dairy and non-dairy food products including yogurts, cottage cheese, pasta, sauces, baked products, cooked meats, and soups. Pediocin PA-1 is also commercially marketed as a fermentate in the form of ALTA^TM^ 2431 by Kerry Group PLC and is chiefly utilized with a view to targeting food-borne pathogens in ready to eat meat products [97]. This commercial fermentate maintains its stability and bioactivity over a range of pH conditions and thus can be utilized to extend the shelf life of a wide range of dairy products as well as other types of foods. Since the pediocin PA-1 producing strain is of GRAS status, addition of fermentates containing this bacteriocin does not require extensive labelling [98].

Danisco has licenced several food-grade cultures and associated defined and undefined fermentates which contain antimicrobial end products to control pathogens in food. Such cultures and undefined fermentates are viewed as more consumer friendly and thus do not require extensive labelling, in contrast to purified fermentates, which require extensive labelling as additives [89,99]. The HOLDBAC^TM^ variety of cultures marketed by Danisco can potentially be applied in meat and dairy products to inhibit the growth of bacterial and fungal pathogens. Other examples include Micocin, marketed by CanBiocin, and this product exhibits antimicrobial activity against *Listeria* in processed meat. Defined antimicrobials such as nisin are marketed as Nisaplin, whereas the product Natamax, produced by fermentation by *Streptococcus natalensis* demonstrates antifungal activity (DuPont). In order to prepare Nisaplin, non-fat milk is initially fermented with a nisin-producing *L. lactis* strain. The fermentate is further spray dried and concentrated and the final product is composed of nisin at 1 × 10^6^ IU/g, 74.4% sodium chloride, 23.8% denatured milk solids, and 1.7% moisture [87,88,89,99]. Overall, Nisaplin is a heat-stable fermentate and is used as a food preservative in a variety of foods such as dairy, meat, bakery products, and beverages, and targets Gram-positive spoilage organisms and pathogens (DuPont). Finally, NovaGARD is another commercial formulation, containing a crude preparation of nisin, produced by DuPont. NovaGARD includes a range of products such as CB1, NR100, NR250, LM100, and 285. These fermentates have different spectra of activity. For instance, products NR100, NR250, and LM100 chiefly target *Listeria* in foods such as cooked and cured meats, fresh soups, and sauces. On the other hand, product CB1 targets Gram-positive pathogens including *Listeria*, while NovaGARD 285 targets Gram-negative organisms and can be used to prolong the shelf life of refrigerated meals, baked goods, soups, and sauces (DuPont).

### 2.6. LAB Fermentates as ACE Inhibitors and Antioxidants

Overall, there has been a dearth of studies evaluating the blood pressure-lowering properties of LAB-derived fermentates. In one such study however, Hayes and co-workers utilized a *Lb. animalis* DPC6134 (NCIMB 41355) strain, which demonstrates proteolytic activity, to generate a bovine sodium caseinate fermentate with a view to assessing the fermentate’s ACE inhibitory activity [100]. Encouragingly, the 10 kDa DPC6134-associated fermentate showed an 85.51% (±15%) ACE-inhibitory activity. In comparison to captopril, which demonstrated an inhibitory concentration (IC_50_) value of 0.005 mg/mL, the DPC6134-associated fermentate demonstrated a 0.8 mg/mL IC_50_ value. Using a combination of membrane filtration and reverse-phase high performance liquid chromatography (RP-HPLC), the authors were able to generate three bioactive fractions. These three bioactive fractions (fractions 10, 19, and 43) demonstrated ACE-inhibitory percentage averages of 67.53, 83.71, and 42.36% respectively. Finally, by employing simulated gastrointestinal digestion conditions, it was shown that the ACE-inhibitory 10 kDa DPC6134 fermentate was recalcitrant to a mix of digestive enzymes including pepsin and corolase PP in the GIT [100].

Fuglsang et al. conducted an in vivo trial investigating the effects of fermentates from up to 26 different LAB strains on ACE inhibition [101]. The study demonstrated that the various synthesized inhibitory substances demonstrated ACE-inhibitory activity in varying concentrations. On the basis of spectrophotometric assays, the authors showed that there was a correlation between peptide formation and ACE-inhibitory ability. Normotensive rats were utilized to ascertain the ACE-inhibitory activity of any such active fermentates. Interestingly, pre-feeding the mice with *Lb. helveticus* fermented milk reduced the pressor effects of angiotensin I (0.3 μg/kg), while one of the fermented milks elicited an elevated response to bradykinin (10 μg/kg) in the normotensive rats. Thus, the authors concluded that the *Lb. helveticus* strains synthesized ACE-inhibitory substances in vivo. While the ACE-inhibitory effect elicited by the *Lb. helveticus* fermentates was relatively low compared to commercial ACE-inhibitors, the fermented milk nonetheless attenuated the conversion of angiotensin I to angiotensin II [101]. The enzyme ACE converts angiotensin I to angiotensin II, and angiotensin II triggers vasoconstriction, and in turn, an increase in blood pressure. The reduced conversion of angiotensin I to angiotensin II, elicited by the fermentates in the study by Fuglsang et al. [101], may result in beneficial anti-hypertensive effects overall [102,103].

A separate study by Daliri et al. investigated the by-products of whey proteins fermented by up to 34 LAB strains, with a particular focus on the ability of the fermentates to suppress ACE enzyme activity [104]. Overall, it was demonstrated that the fermentates varied in their ability to suppress the enzymes in vitro. There were seven promising fermentates which demonstrated potent ACE-inhibitory activity in the range of 52.40%–84.7% and IC_50_ values ranging from 2.13–19.78 mg/mL. The strain which possessed the most potent ACE-inhibitory activity was *Pediococcus acidilactici* SDL1414, with a value of 84.7% ACE-inhibition and IC_50_ value of 19.78 mg/mL. Interestingly, it was shown that up to 57.7% of the peptides <7 kDa produced by this strain inhibited ACE. The strain SDL1414 may have potential as a starter culture in the dairy industry with a view to being used to control hypertension [78,104].

In relation to the antioxidant activities associated with LAB-derived fermentates, Virtanen and co-workers assessed the fermentates from several LAB strains grown in milk [105]. Out of the 25 LAB strains evaluated in the study, fermentates from 6 strains demonstrated potent radical scavenging ability, of which *Lactobacillus jensenii* ATCC 25258, *Lb. acidophilus* ATCC 4356, and *Leuconstoc mesenteroides* subsp. *cremoris*-derived fermentates showed the highest level of antioxidant activity. Overall, the lipid peroxidation inhibition activity of fermentates from these strains was weaker than the radical scavenging ability. In four of these six strains, the radical scavenging activity was linked with proteolytic activity. It was found that there was a correlation between potent radical scavenging ability and a larger percentage of peptides ranging from 4–20 kDa found in the fermentates, and in general, there was a higher proportion of hydrophobic amino acids present in these fermentates. Overall, the antioxidative capabilities of the fermentates were strain specific and there was an association between the synthesis of radical scavengers and simultaneous proteolytic activity of the strains. On the other hand, lipid peroxidation ability correlated with the degree of bacterial growth [105].

### 2.7. Effects of LAB Fermentates on Cognitive Health

In a recent study by Warda et al., the authors conducted a mouse trial and evaluated the impact of feeding the mice a heat-killed fermentate composed of two *Lactobacillus* strains, with a particular focus on impacts on host behavior and alterations in the host microbiota [106]. Encouragingly, mice which were fed this fermentate (ADR-159), showed greater levels of sociability and had reduced baseline corticosterone levels. The fermentate also resulted in minor alterations in the mouse microbiota and impacted the less abundant taxa more dramatically than the dominant taxa. Furthermore, it was encouraging that the fermentate did not elicit any undesired adverse effects on anthropometrics and overall health of the mice [106]. ADR-159 was a co-fermentate of one *Lactobacillus delbrueckii* and one *Lactobacillus fermentum* strain, along with the culture medium (which is composed of lactose monohydrate, yeast extract, dipotassium phosphate, casein peptone, and sodium acetate). The fermentate underwent extended high temperature treatment after production and there were approximately 6 × 10^10^ viable bacterial cells present per gram of ADR-159 fermentate. ADR-159 was a commercial product synthesized by Adare Pharmaceuticals SAS. As part of the animal trial, the final concentration of the ADR-159 fermentate incorporated into standard mice chow was 5%, which resulted in approximately 3 × 10^9^ cells per gram of chow [106].

A summary of patents and publications pertaining to the health benefits elicited by LAB fermentates is included in Table 1 and Table 2, respectively.

## 3. Conclusions

In this mini-review, we have summarized some key studies and patents relating to the diverse range of health benefits conferred by LAB-derived fermentates. As mentioned, such fermentates can have positive impacts on gut health, as well as overall general health. In many cases, the precise mechanisms of action responsible for the health benefits exerted by these fermentates have not been elucidated, and thus further in vitro, in vivo, and clinical trials are warranted to fully unravel such complex mechanisms. Any such insights into the mode of action of such fermentates may reveal whether the bacterial cells have to be active or whether the effects are merely mediated by cell surface components and/or metabolites and bioactives present in the culture supernatants/fermentation media. Indeed, the health benefits associated with biofunctional fermented foods can be exerted either by the live microbes present in the foods themselves directly (probiotic effect) or via the metabolites and bioactive products that the live microbes produce (bioactive effect) [11,107,108,109]. Furthermore, recent advances in next generation sequencing technology can be harnessed to gain deeper insights into the impact that LAB and fermentates thereof can have on the overall gut microbiota. Since there is a growing body of evidence showing links between gut microbiota composition and overall health, the gut microbiota can potentially be beneficially modulated by such LAB fermentates in a target-specific manner. Although it is beyond the scope of this review, it must be noted that prebiotics represent another important avenue with respect to beneficially modulating the gut microbiota. Indeed, a number of studies and reviews have highlighted the ability of prebiotics such as inulin, fructo-oligosaccharides (FOS), and galact-oligosaccharides (GOS) to modulate the gut microbiota, thereby leading to improvements in gut health and overall health [110,111,112,113,114,115,116,117,118]. The primary focus of this review however has been about the overall health benefits, including gut health benefits, derived from LAB fermentates.

## Figures and Tables

**Table 1:** Patents relating to health benefits of Lactic Acid Bacteria (LAB) fermentates.

Patent Inventors, Application Number and Patent Number	Patent Title	Strain(s) and Associated Fermentates	Health Effects
Francesca Romana Fasano and Andrea Luigi Budelli (H.J. Heinz Company Brands LLC)(18157990.5) (EP 3 351 554 A1)	Gluten-Related Disorders	*Lb. paracasei* CBA L74 metabolites	Improved gluten-associated disorders. Elicited reduced influx of gliadin peptides into cells.
Andrea Budelli, Francesca Romano Fasano, Miryam Terzano, and Lorenzo Bramati (H.J. Heinz Company brands LLC) (18166007.7) (EP 3 366 143 A1)	Probiotic Compositions and Methods	*Lb. paracasei* CBA L74 replicating or non-replicating form. Metabolites (including acids) of *Lb. paracasei* CBA L74 strain in a replicating or non-replicating form in fermented foods.	Modulate mucosal immune system and improved gut disorders. Induced mucosal immune system, excluded pathogens, ameliorated enteral nutrition.
Francesca Romana Fasano and Maria Rescigno (H.J Heinz Company Brands LLC) (18209662.8)(EP 3 470 074 A1)	Probiotics and Methods of Use	Foods fermented with *Lb. paracasei* CBA L74 in a replicating or non-replicating form (results based on mouse trial).	Reduced severity of an infection in offspring of lactating mice. Effective against several pathogens including *Listeria monocytogenes*, *Salmonella typhimurium*, and *Cronobacter sakazakii*.

**Table 2:** Publications relating to health benefits of LAB fermentates.

LAB Strains and Associated Fermentates	Effects	Fermentation Substrate	Reference
*Lb. helveticus* fermentates	Stimulated GLP-1 production, anti-diabetic effect	11% RSM	75
Fermentates from a range of LAB strains	Inhibited pancreatic lipase.	10% RSM	71
Fermentates of *Lactobacillus pentosus* DPC6004, *Lactobacillus salivarius* subsp. *salivarius* DPC6005, *Pediococcus pentosaceus* DPC6006, *Lactobacillus murinis* DPC6002, and *Lb. murinis* DPC6003	Anti-*Salmonella* effect in pigs	All strains in 10% RSM with yeast extract except *Lb. salivarius* (10% RSM only without yeast extract).	83
*Lb. acidophilus* SBT2074	Inhibited *Escherichia coli* 3544	10% RSM	84
Fermentates of *Lb. animalis* DPC6134 (NCIMB 41355)	ACE inhibitory activity	2.5% sodium caseinate substrate with 0.5% glucose	100
26 LAB fermentates	ACE inhibitory activity	9.5% RSM	101
34 LAB fermentates. Most potent was *Pediococcus acidilactici* SDL1414	ACE inhibitory activity	20% Soybean protein isolates	104
Fermentates from *Lactobacillus jensenii* ATCC 25258, *Lb. acidophilus* ATCC 4356, and *Leuconstoc mesenteroides* subsp. *cremoris*	Antioxidant activity	Fresh pasteurized skim milk	105
Fermentates from *Lb. delbrueckii and Lb. fermentum* (ADR-159)	Mice demonstrated increased sociability and decreased baseline corticosterone	Culture medium containing lactosemonohydrate, casein peptone, yeast extract, sodium acetate, dipotassiumphosphate).	106
